# The association between sleep quality and cognitive function in patients with non-functioning pituitary adenoma

**DOI:** 10.1530/EC-25-0082

**Published:** 2025-09-23

**Authors:** M Brown, I L Ross, W Nkoana, M Henry

**Affiliations:** ^1^ Department of Psychology, University of Cape Town, Rondebosch, South Africa; ^2^ Department of Endocrinology, University of Cape Town, Rondebosch, South Africa; ^3^ Department of Psychology, School of Human and Community Development, University of the Witwatersrand, Johannesburg, South Africa; ^4^ Centre for Higher Education Development, University of Cape Town, Rondebosch, South Africa

**Keywords:** pituitary disease, non-functioning pituitary adenomas, cortisol, growth hormone, sleep, memory

## Abstract

**Background:**

Cortisol and growth hormone are important for sleep regulation and cognition. Sleep is critical for cognitive functioning and memory consolidation. Patients with pituitary disease experience hormonal dysregulation, impaired sleep quality, and cognitive dysfunction. We wished to examine the relationship between objective sleep and cognitive functioning in patients with pituitary adenomas.

**Methods:**

Ten patients with non-functioning pituitary adenomas (NFPA) and ten healthy controls were assessed using a crossover design. Each participant was administered standardised neuropsychological tests (Wechsler logical memory test (LMT) and finger tapping task (FTT)) assessing declarative and procedural memory performance after a period of sleep and after an equivalent period of wakefulness. Objective sleep data were elicited, and the Pittsburgh Sleep Diary captured self-reported sleep data.

**Results:**

Controls performed better than patients with NFPA on retention on the LMT (*P* = 0.027). Objective measures of sleep quality revealed no between-group differences, whereas controls reported better subjective sleep quality (*P* = 0.016) and being more alert when awake (*P* = 0.015) than patients. Generally, sleep was not related to cognition in either group.

**Conclusions:**

Patients demonstrated poorer performance on declarative memory tasks, but not poorer sleep. Interventions for memory rehabilitation may assist their capacity to complete other important daily activities.

## Background

Sleep is vital for overall health and well-being (1), playing crucial roles in promoting cognitive functioning, particularly in the domain of memory consolidation (2, 3). When people experience impaired sleep, they perform more poorly on memory tests than those whose sleep is uninterrupted (4, 5).

Growth hormone (GH) and cortisol secretion are closely linked to the sleep–wake cycle (6). Secretion and inhibition of cortisol play key roles in regulating sleep onset, where it is at its nadir, and at termination of sleep, where it is highest, respectively. Night-time awakenings are accompanied by an initial release of corticotropin-releasing hormone and cortisol, and subsequently by the temporary inhibition of these hormones. The relationship between the hypothalamic-pituitary-adrenal axis and sleep is bidirectional: cortisol directly impacts sleep, and sleep directly inhibits cortisol (7, 8). Davidson *et al.* (9) examined the secretory patterns of GH and cortisol in relation to sleep and wakefulness. During sleep deprivation, the GH surge disappeared, whereas during recovery sleep GH excursions were greater and the secretion was prolonged, indicating that the GH surge shifts with the sleep–wake cycle and that the nocturnal GH surge is largely sleep-dependent.

GH interacts with brain structures and affects cognitive functioning (10). GH receptors are concentrated in the hippocampus, which is critical for learning and memory (10, 11). GH excess is associated with impairment of attention, visuoconstructional ability, memory, verbal fluency, and executive function (12). GH replacement therapy administered to deficient individuals results in enhanced mental alertness, attention, motivation, and memory (13). The relationship between cortisol and cognitive performance follows an inverted-U shape. To achieve optimal cognitive function, a moderate amount of cortisol is needed; concentrations that are too low or too high have negative effects on cognition (14). Evidence suggests that both exogenously supplemented and endogenous secretion of cortisol have marked effects on hippocampal-dependent cognition, especially learning, encoding, and recall of declarative memory material (15, 16). The hippocampus has high concentrations of mineralocorticoid and glucocorticoid receptors, with the former being important in appraisal of incoming material, encoding of information, and memory retrieval, whereas the latter complements this by promoting memory consolidation and behavioural reactivity (17).

Pituitary disease (PD) is commonly caused by a tumour of the pituitary gland. Patients with these tumours may require various lifelong hormone replacement therapies. Pituitary tumours are treated with i) transsphenoidal resection, ii) hormone replacement therapy, iii) radiotherapy, or iv) a combination. The course of treatment is dependent on the size of the tumour and the symptoms (18, 19).

Studies indicate that patients with PD secondary to non-functioning pituitary adenomas (NFPA’s) experience compromised sleep quality. Polysomnographic and actigraphy data from patients with non-functioning pituitary macroadenomas (NFMA) reported reduced sleep efficiency, less REM sleep, more night-time awakenings, longer sleep duration, and less daytime activity relative to healthy controls (HCs). Patients also self-reported excessive fatigue and sleep disturbance (19). In Cushing’s syndrome (a disorder characterised by an overproduction of cortisol), patients frequently experience poor sleep quality, characterised by nocturnal fragmentation, increased wrist movements, longer time spent awake after sleep onset, and reduced slow-wave sleep (20, 21). Associations between GH and sleep in patients with acromegaly (a PD characterised by the overproduction of GH) indicate that they manifest with sleep apnoea and reduced self-perceived and subjective sleep quality (22, 23).

Cognitive dysfunction is a major complication of PD (24, 25, 26). Yedinak and Fleseriu (27) showed that patients with acromegaly self-reported poor learning, concentration, and difficulty maintaining focus on imminent tasks, whereas patients with NFPA self-reported cognitive dysfunction, relative to mental agility and verbal memory recall.

As patients with PD experience fluctuations in hormone secretion, due to endogenous imbalances or by exogenous supplementation of hormones, and often report poor sleep patterns and poor memory, they are an ideal group to study sleep quality and cognitive function.

We wished to investigate whether sleep enhances memory consolidation in patients with NFPA to the same degree as it does in controls, hypothesising that, compared to matched HCs, patients with PD will have poorer sleep quality and will perform more poorly on standard tests of memory. We also hypothesise that both patients and controls will have better memory performance when a period of sleep, rather than a period of wakefulness, precedes testing of recall.

## Participants

### Power analysis

G*Power software (28) indicated that to achieve statistical power >0.90 using a repeated-measures ANOVA investigating between- and within-group differences and parameters set at correlation among repeated measures = 0.50 and effect size (Cohen’s *f*) = 0.25, a sample of *n* = 18 (*n* = 9 per group) would be required.

### Recruitment

**Figure 1:**
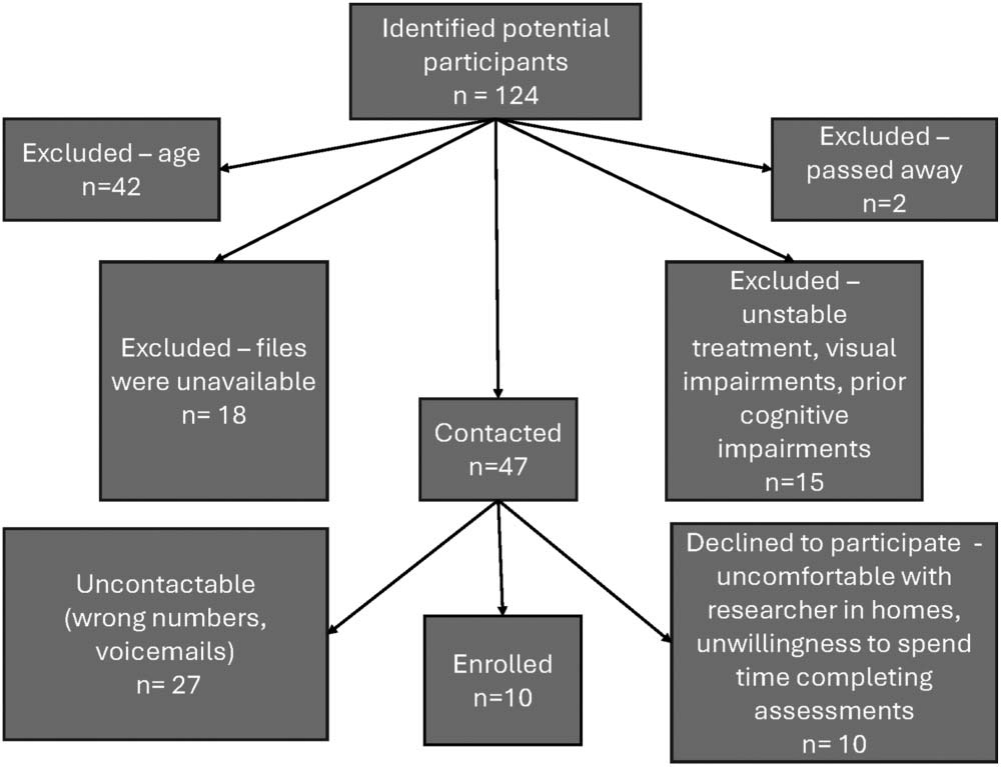
Flowchart depicting the patient recruitment process. Of the 124 potential participants identified, 42 were excluded due to age, 18 due to unavailable files, 15 due to unstable treatment, visual impairments, or prior cognitive impairments, and 2 had passed away. A total of 47 participants were contacted. Of these, 27 were uncontactable, 10 declined to participate, and 10 were enrolled in the study.

We recruited ten adult patients (seven women, three men) diagnosed with an NFPA from the pituitary clinic. Our analyses (independent sample *t*-tests and chi-square) detected no significant difference in the patients enrolled compared to the remaining eligible patients in the database in terms of age, gender, endocrine type, and therapy. Although the patients who enrolled in our study were relatively few, they did not differ from the remaining patients, suggesting that there may be external validity for our findings. Eligible patients were called, informed about the nature of the study, and invited to participate. Figure 1 is a flowchart depicting in detail the steps taken in this recruitment process. We enrolled ten HC subjects who were case-matched for age (within 3 years), level of education (within 2 years), and sex. HCs were recruited following adverts on social media informing eligibility criteria.

### Eligibility criteria

As sleep architecture and cognitive function are affected by age, especially in children and the elderly (29), only individuals 18–65 years of age were eligible to participate. Individuals prescribed medication known to have sedative properties (e.g. sedatives and anxiolytics) were excluded. Individuals with an IQ lower than 85 on the Shipley IQ test were excluded. All participants required a basic level of English fluency, as all study instruments were in English. Pregnant women were excluded, as they are prone to variations in sleep patterns (30). Participants with a history of i) neurological disorders that could affect cognitive function (e.g. dementia, epilepsy, severe head injury, stroke), ii) known sleep disorders (including sleep apnoea), or iii) psychiatric illness associated with disrupted sleep and/or cognition (31), were excluded.

Patients were on stable replacement therapy for at least 3 months before study enrolment. HCs had to be free of chronic illness and not using any chronic medication.

### Study design

We used a quasi-experimental within-subjects repeated-measures design. The independent variables (IVs) were group (patients with PD and HCs) and experimental condition (*sleep* and *wake*). Regarding the second IV, each participant experienced a sleep condition (learning and recall of material were separated by 12 h of sleep) and a *wake* condition (learning and recall separated by 12 h of daytime wakefulness), with a crossover design. The two protocols were separated by 1 week, during which participants were required to wear a lightweight wristband (Fitbit Alta HR, Fitbit, Inc., USA) to track sleep patterns and to keep a daily sleep diary. The dependent variables were: i) objectively measured memory performance and ii) sleep quality. All screening and testing took place in participants’ homes. Patients and controls were assessed within 7 days of each other, to limit differences in hours of daylight and timing of the sunrise and sunset variations, which could have influenced sleep patterns.

### Screening measures

A study-specific Sociodemographic and Medical Questionnaire determined study eligibility, including biographical and medical information (e.g. age, sex, level of education, clinical history, and treatment regimen) from participants.

The Mini International Neuropsychiatric Interview (English version 5.0.0; MINI; (32)) is a brief interview-based diagnostic instrument that was used to screen for the presence of a major DSM-IV (33) Axis psychiatric disorder.

The Beck Depression Inventory-Second Edition (BDI-II; (34)) is a brief instrument measuring intensity and depth of depression in adolescents and adults. Individuals who scored >29 were excluded from participation.

The Shipley-2 Intelligence Test (35) is a brief measure of general intellectual functioning, requiring completion of two multiple-choice subtests. An overall IQ score can be derived from aggregating the vocabulary and the block patterns scales.

### Memory tests

The logical memory (LM) subtest of the Wechsler Memory Scale-third revision (WMS-III; (36)) measures narrative episodic memory. The test administrator reads a short story and then asks the participant to repeat it as accurately as possible; this is repeated for a second story. In this study, an additional set of recall trials (one for each of the two stories) was administered after 12 h. We used four short stories in total: two were presented in the sleep–wake condition and the remaining two in the wake–sleep experimental condition. Hence, every participant (patient and control) heard the same stories in the same condition.

The finger tapping task (FTT; (37)) is a computerised task assessing procedural memory. Participants are required to use their non-dominant hand to repeatedly type, for 30 s, a 5-element number sequence. The sequence always appears at the top of the screen to avoid reliance on working memory. This procedure is repeated for 12 trials, with each trial i) presenting a sequence and ii) separated from the next by a 30 s break. Performance is scored as the number of correctly repeated sequences on each trial. In this study, the participants were asked to complete three more 30 s trials after 12 h.

To eliminate the possibility of practice effects, different forms of the LM and the FTT were used in the *sleep* and *wake* conditions. In addition, to limit the possibility that multiple investigators could have had an impact on the results of the study, there was only one investigator who performed the same research on both the patients and controls in all cases.

### Assessments of sleep quality

The Fitbit Alta HR recorded activity and sleep patterns in everyday environments during the *sleep* and *wake* conditions, and in the intervening week between the two conditions. The Fitbit Alta HR is a lightweight wristband, which records motion data and discriminates whether the participant is asleep or awake.

The Pittsburgh Sleep Diary (38) collected self-reported data on sleep patterns (e.g. time of onset of sleep and time of awakening) overnight during the *sleep* condition and during the week between the *sleep* and *wake* conditions.

### Deriving outcome variables

We scored the two memory tests following standard procedures (36, 37). The LM outcome variables were: i) learning, the sum of all elements recalled correctly across story A and story B; and ii) percent retention, the number of story elements recalled correctly in the delayed recall of story A and story B, divided by the number of story elements recalled during learning. The FTT outcome variables were: i) post-training performance, the average number of completed sequences across the last three trials of the training session; ii) post-training error rate, the average number of errors across the last three trials of the training session; and iii) percent retention, the average number of completed sequences across the three retest trials divided by post-training performance.

Data from sleep diaries informed when participants were sleeping, when they got into bed at night, when they woke up in the morning, and subjective sleep quality. The Fitbit provided improved data about their actual sleep. From these two data sources, we derived for the sleep condition night: i) sleep latency, number of minutes between turning out the lights at night and falling asleep (sleep onset); ii) total sleep time (TST), number of minutes spent sleeping between sleep onset and morning waking; iii) wake after sleep onset (WASO), number of minutes spent awake between sleep onset and morning waking; iv) sleep efficiency, TST divided by time in bed multiplied by 100; and v) number of awakenings, number of times the participant woke up for more than 1 min between sleep onset and morning waking. Subjective sleep variables (derived from the sleep diary), in which they rated aspects of their sleep on a scale from 1 to 10, were: i) sleep quality, 1 being very bad and 10 being very good; ii) mood upon awakening, 1 being very tense and 10 being very calm; and iii) alertness upon awakening*,* 1 being very sleepy and 10 being very alert.

### Procedure

After participants read and signed informed consent documents, eligibility was confirmed after the screening measures described above. Each patient and their matching control were randomly assigned to either the sleep–wake or the wake–sleep administration sequence. Hence, half of the participants (wake–sleep sequence) experienced the wake condition first and the other half (sleep–wake group sequence) experienced the sleep condition first. We randomised the patients into either the sleep–wake or wake–sleep experimental condition. The controls then experienced the conditions in the same order as their matched patient. In the *wake* condition, participants were instructed not to sleep between the morning and evening memory testing sessions. In the *sleep* condition, participants were instructed not to sleep during the day ahead of the evening testing session. All participants were encouraged to maintain their usual daily routines. Patients and controls were assessed within seven days of each other, to limit differences in hours of daylight and timing of the sunrise and sunset variations, which could have influenced sleep patterns.

**Figure 2:**
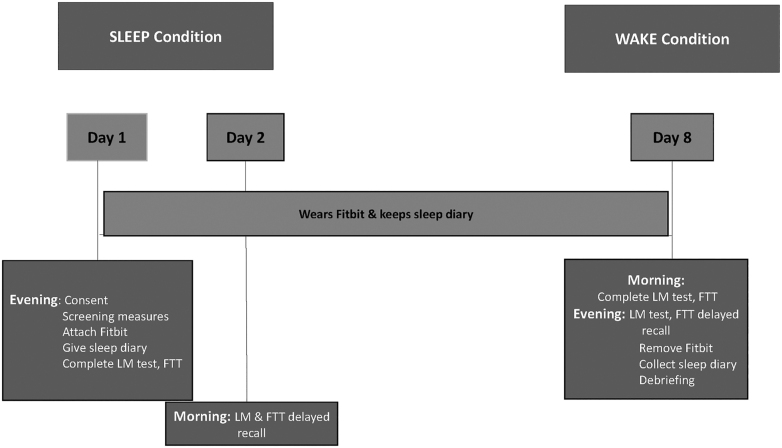
Study protocol for the sleep and wake conditions. Researchers arrived at participants’ homes at 19:00 h. Each participant was provided with a sleep diary, Fitbit, and instructions, and subsequently completed the LM learning and immediate recall trials and the initial set of FTT trials. They completed the delayed recall and re-test trials 12 h later (i.e. the next morning). After a week, the counterbalanced procedure was administered.

**Figure 3:**
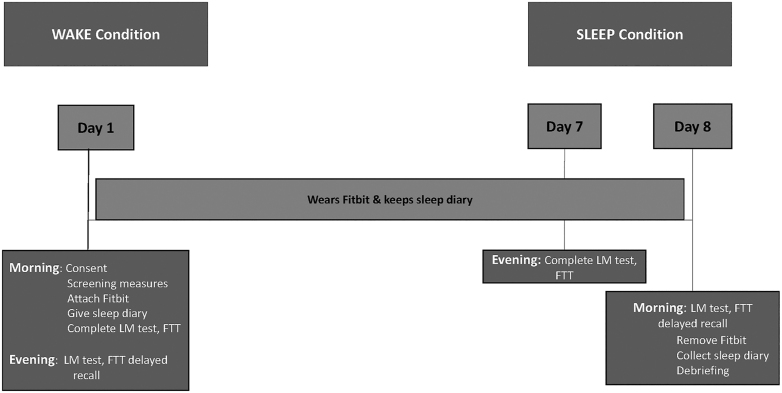
Study protocol for the wake–sleep condition. Researchers arrived at participants’ homes at 07:00 h. Each participant was provided with a sleep diary, Fitbit, and instructions, and subsequently completed the LM learning and immediate recall trials and the initial set of FTT trials. They completed the delayed recall and re-test trials 12 h later (i.e. the same day in the evening). After a week, the counterbalanced procedure was administered.

Researchers arrived at participants’ homes at either 19:00 h (*sleep* condition; see Fig. 2) or 07:00 h (*wake* condition; see Fig. 3), and each participant was provided with a sleep diary, Fitbit, and instructions, and subsequently completed the LM learning, immediate recall trials, and the initial set of FTT trials. They completed the delayed recall and retest trials 12 h later (i.e. the next morning if they were in the *sleep* condition, or the same day in the evening if they were in the *wake* condition). After a week, the counterbalanced procedure was administered.

### Ethical considerations

Ethical approval was obtained from the University of Cape Town (UCT) Department of Psychology Research Ethics Committee, from the UCT Faculty of Health Sciences Human Research Ethics Committee, and from Groote Schuur Hospital’s Research Ethics Committee.

### Data management and statistical analyses

We used SPSS (version 26.0) to complete all analyses, with the threshold for statistical significance set at α = 0.05. Examination of q–q plots indicated there were no significant outliers in the data and that none of the assumptions underlying any of the inferential statistical analyses described below were violated.

### Inferential analyses

Analyses followed four discrete steps. First, independent sample *t*-tests (for continuous variables) and chi-squared tests (for categorical variables) assessed the magnitude of between-group differences relative to sociodemography, depression, and IQ. A series of independent sample *t*-tests assessed the magnitude of between-group differences regarding variables indexing objective and subjective sleep quality on the night of the sleep condition. A series of 2 × 2 (group × condition) repeated-measures ANOVAs assessed between- and within-group differences on the LM and FTT outcome variables. Fourth, a series of bivariate correlational analyses assessed whether, within each group, sleep outcome variables were associated with cognitive outcome variables.

## Results

### Sample characteristics

The age range of the participants was 29–65 years (mean = 50.55, SD = 11.84), having completed between 8 and 13 years of formal education (mean = 11.10, SD = 1.46), and most (70%) were women. Analyses detected no between-group differences in age, gender distribution, education, depression, and general intellectual functioning (see Table 1). All participants scored in the ‘minimal or no depression’ range (0–13 points; (34)). These results are not expected to influence either sleep or cognition (39, 40).

**Table 1:** Sociodemographic characteristics (*n* = 20).

Variable	Group		*t*/*χ*^2^	*P*	ESE
	NFPA patients	HCs			
	(*n* = 10)	(*n* = 10)			
Age (years)			−0.20	0.838	−0.09
Mean (SD)	51.10 (11.29)	50.00 (12.39)			
Range	29–65	31–65			
Education (years)			0.60	0.551	0.27
Mean (SD)	10.90 (1.37)	11.30 (1.56)			
Range	8–12	8–13			
Sex			0.00	1.00	0.00
Male (*f*, %)	3 (30.00)	3 (30.00)			
Female (*f*, %)	7 (70.00)	7 (70.00)			
BDI-II total score					
Mean (SD)	3.60 (1.77)	2.50 (2.75)	1.06	0.303	0.47
Range	1–6	0–9			
Shipley-2 IQ score			0.42	0.674	0.19
Mean (SD)	93.60 (13.84)	91.50 (7.02)			
Range	73–123	86–104			

ESE, effect size estimate (Cohen’s *d* for *t*-tests and Cramer’s *V* for chi-square tests); *f*, frequency; HCs, healthy controls.

### Patient clinical characteristics

Patients had been diagnosed at an average age of 42 years and disease duration of 9 years. Most of our patients (70%) underwent radiotherapy and surgery, with the remainder only having undergone transsphenoidal surgery (see Table 2). All patients who received radiotherapy received it more than a year previously. By virtue of the inclusion criteria, all patients had been on a stable treatment regimen for at least 3 months before enrolment. The following hormone replacement regimens were used: hydrocortisone (*n* = 6), L-thyroxine (*n* = 9), depot testosterone (*n* = 2), and oestrogen replacement (*n* = 4). In addition, two patients had GH deficiency, both of whom were not supplemented, and one patient had arginine vasopressin deficiency. The associated comorbidities are also shown in Table 2.

**Table 2:** Demographic and clinical data of enrolled patients with non-functioning pituitary adenomas (*n* = 10).

Variable	Statistic	Combination deficiency	Statistic
Gender, female	7 (70%)		
		**Number of endocrine deficiencies**	
Age at diagnosis (years)		1 (*f*, %)	3 (30%)
Mean (SD)	41.90 (9.58)	2 (*f*, %)	1 (10%)
Range	24–50	3 (*f*, %)	1 (10%)
		4 (*f*, %)	1 (10%)
		5 (*f*, %)	1 (10%)
Duration of disease (years)		**Treatment**	
Mean (SD)	9.20 (5.61)	Surgery (*f*, %)	10 (100%)
Range	3.20	Radiotherapy	7 (70%)
		Surgery and radiotherapy	7 (70%)
**Endocrine deficiencies**		**Replacement therapy**	
Secondary hypothyroidism (*f*, %)	9 (90%)	Hydrocortisone (*f*, %)	6 (60%)
Secondary hypoadrenalism (*f*, %)	7 (70%)	L-thyroxine (*f*, %)	9 (90%)
Hypogonadotrophic hypogonadism (*f*, %)	6 (60%)	Depot testosterone (*f*, %)	2 (20%)
GH deficiency (*f*, %)	2 (20%)	Oestrogen replacement (*f*, %)	4 (40%)
Arginine vasopressin deficiency (*f*, %)	1 (10%)	GH replacement (*f*, %)	0 (0%)
		Desmopressin (*f*, %)	1 (10%)
**Medical comorbidities**		**Cases**	
Type 2 diabetes mellitus (*f*, %)	3 (30%)	i) Hypothyroidism (lone)	3 (30%)
Hypertension (*f*, %)	1 (10%)	ii) Hypothyroidism and hypoadrenalism	3 (30%)
Eczema (*f*, %)	1 (10%)	iii) Hypothyroidism, hypoadrenalism, hypogonadism	1 (10%)
Gout (*f*, %)	1 (10%)	iv) Hypothyroidism, hypoadrenalism, hypogonadism and GH deficiency	1 (10%)
Dyslipidaemia (*f*, %)	2 (20%)	v) Hypothyroidism, hypoadrenalism, hypogonadism, GH deficiency, AVPD	2 (20%)
Ischaemic heart disease (*f*, %)	1 (10%)		
Folate deficiency (*f*, %)	1 (10%)		
Pulmonary tuberculosis (*f*, %)	1 (10%)		

*f*, frequency; GH, growth hormone.

### Sleep quality on the sleep night: between-group comparisons

Analyses detected no between-group differences on any of the objectively measured sleep outcome variables (all *P* > 0.114). Between-group differences were found in sleep quality, alertness upon awakening, and a trend towards significance regarding mood upon awakening (see Table 3). Controls rated their sleep quality more positively, reported greater alertness, and better mood upon awakening than patients.

**Table 3:** Sleep quality over the night of the sleep condition: descriptive statistics and between-group comparisons (*n* = 20).

Variable	Group		*T*	*P*	ESE
	NFPA patients	HCs			
	(*n* = 10)	(*n* = 10)			
Objective measures					
Sleep latency (min)	22.50 (28.98)	27.40 (21.75)	0.42	0.674	0.16
TST (min)	371.20 (93.30)	384.30 (62.20)	0.36	0.716	0.19
WASO (min)	34.10 (17.95)	36.80 (23.39)	0.28	0.776	0.12
Sleep efficiency (%)	0.91 (0.04)	0.91 (0.05)	−0.11	0.911	−0.05
Number of awakenings	2.10 (1.44)	1.30 (0.48)	−1.65	0.115	−0.74
Subjective measures					
Sleep quality	7.01 (2.23)	8.90 (1.11)	−2.38	0.016*	−1.06
Alertness upon waking	7.30 (2.29)	9.27 (1.35)	−2.36	0.015*	−1.05
Mood upon waking	7.92 (2.26)	9.28 (1.20)	−1.67	0.056	−0.74

In the second and third columns, data is presented as mean (SD). ESE, effect size estimate (in this case, Cohen’s *d*); HCs, healthy controls.

All *P*-values are one-tailed, given that analyses were testing the hypothesis (based on the previous literature) that sleep quality would be better in controls than in patients. Statistically significant *P*-values are denoted in boldface font.

* *P* < 0.05.

### Cognitive data: effects of group, condition, and the group × condition interaction

Table 4 presents descriptive data on LM and FTT performance for both patients and controls within the sleep and wake conditions.

**Table 4:** Cognitive data: performance by patients and controls in the sleep and wake conditions (*n* = 20).

Variable	Group			
	NFPA patients		HC	
	(*n* = 10)		(*n* = 10)	
	Sleep	Wake	Sleep	Wake
LM				
Learning	22.20 (4.44)	24.70 (5.41)	24.50 (4.19)	23.00 (7.51)
Percent retention	72.66 (21.09)	66.73 (18.46)	88.44 (14.80)	82.22 (19.58)
FTT				
Post-training performance	20.44 (12.83)	22.00 (13.49)	28.90 (10.57)	30.70 (10.30)
Post-training error rate	0.01 (0.02)*	0.06 (0.12)*	0.01 (0.01)	0.02 (0.01)
Percent retention	91.01 (20.39)	86.23 (25.23)	94.81 (48.61)	89.74 (17.55)

Data is presented as mean (SD).

* Data based on nine participants (one patient did not complete the FTT).

HCs, healthy controls; LM, logical memory; FTT, finger tapping task.

#### Declarative memory: LM test

Regarding the learning variable, the analysis detected no significant main effect of condition, *F*(1.18) = 0.22, *P* = 0.644, *η*^2^ < 0.001, or of group, *F*(1.18) = 0.018, *P* = 0.895, *η*^2^ < 0.01, but it detected a trend towards a significant group × condition interaction, *F*(1.18) = 3.53, *P* = 0.076, *η*^2^ = 0.03. Within-group post-hoc pairwise comparisons, we found a trend towards a significant between-condition difference for the patient group (*P* = 0.057; better performance in the wake than in the sleep condition, as shown in Table 4), but no trend within the control group (*P* = 0.166; relatively equivalent performance in the two conditions, as shown in Table 4).

Regarding the percent retention outcome variable, the analysis detected a significant main effect of group, *F*(1.18) = 5.80, *P* = 0.027, *η*^2^ = 0.16, but no significant main effect of condition, *F*(1.18) = 1.35, *P* = 0.260, *η*^2^ = 0.02, and no significant group × condition interaction, *F*(1.18) = 8.20, *P* = 0.977, *η*^2^ < 0.01. The significant effect of group indicates that, regardless of experimental condition, controls retained more information in the interval between learning and recall than patients (see Table 4). Although this difference did not reach significance, both patients and controls retained more information when a period of sleep, rather than a period of wakefulness, separated learning and recall.

#### Procedural memory: FTT

The analyses detected no main effect of group, no significant main effects of condition, and no significant group × condition interactions for any FTT variables, all *P* > 0.120.

### Within-group associations between sleep and cognitive variables

Within the NFPA patient group, there were no correlations between sleep and cognitive variables, all *P* > 0.070, except for sleep efficiency and FTT post-training error rate, *r* = −0.817, *P* = 0.007. Better sleep efficiency was associated with a smaller FTT post-training error rate.

**Figure 4:**
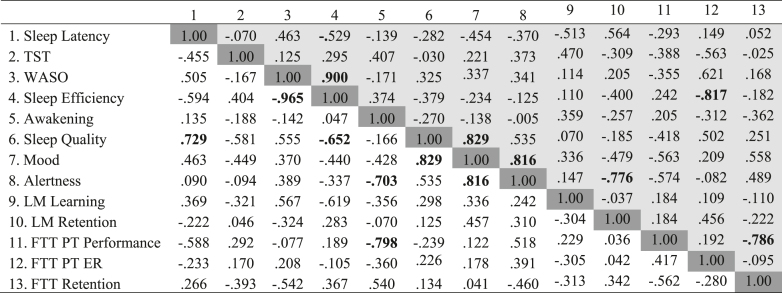
Bivariate correlations for the NFPA patient group and HC groups: associations between sleep outcome variables and cognitive outcome variables (*n* = 20). Note. Data presented are Pearson’s r correlation coefficients. Patient data are presented in the top right cells of the table, shaded light grey; control data are presented in the bottom left cells of the table, not shaded. Statistically significant associations (one-tailed) are highlighted in boldface font.

Within the HC group, there were no correlations between sleep and cognitive variables, all *P* > 0.074, except for awakenings and FTT post-training performance, *r* = −0.798, *P* = 0.006 (see Fig. 4). Fewer awakenings were associated with better post-training performance on the FTT.

## Discussion

Our study aimed to describe sleep quality, memory, and their relationships in patients with PD relative to HCs. Controls’ retention of verbal declarative materials was significantly better than that of patients. However, there were no significant between-group differences in objectively measured sleep quality.

### Hypothesis 1: between-group differences in sleep quality and cognitive functioning

It was hypothesised that compared to HCs, patients would have poorer sleep quality, which was not confirmed, as there were no between-group differences on any of the objectively monitored sleep variables, except for non-significant trends suggesting that patients had shorter TST and more night-time awakenings.

These non-significant findings with respect to the sleep quality of patients with PD, in comparison to controls, are in contradistinction to existing data (20, 41, 42). Previous studies reported that patients with NFMA experience significantly compromised sleep quality as opposed to our study, potentially explained by the fact that in previous studies polysomnography was used, whereas we utilised the Fitbit Alta HR*,* with the latter being less sensitive to detect subtle changes in sleep quality than polysomnography (PSG) and actigraphy (41, 42).

Although the Fitbit Alta HR has been shown to be a viable alternative to actigraphy, as it can reliably differentiate between sleep and wake states and provide gross estimates of sleep parameters, it has limitations (43, 44). Albeit that the number of participants is small, one must consider that our findings could be a true reflection of the lack of significant differences in sleep quality. Van der Klaauw *et al.* (45) found that patients with NFMA experienced normal sleep patterns (onset, sleep timing, duration, and rise time), but they also reported significant daytime sleepiness. Our sample of NFPA patients reported significantly poorer sleep quality, decreased levels of alertness upon awakening, and lower mood upon awakening compared with controls.

Lipinska & Thomas (46) found differences between objectively measured and perceived sleep quality in individuals diagnosed with post-traumatic stress disorder, suggesting that subjective reports of sleep quality do not correlate with objective measures. It is unknown whether significant impairments in PD patients’ sleep architecture, study design, or methodology can account for objective and perceived sleep differences. Biermasz *et al.* (20) showed that NFMA patients experience decreased REM sleep and increased stage 1 sleep, which we could not replicate, suggesting that it may be a function of the insensitive Fitbit we utilised, warranting laboratory-based PSG to define sleep stages more accurately.

The second part of hypothesis 1 stated that patients with NFPA would have worse cognitive function than matched HCs, which was partially confirmed, with patients performing significantly more poorly on some (but not all) tasks assessing verbal declarative memory and procedural memory. Specifically, on a story memory task, controls recalled more information than patients. Regarding procedural memory, controls made fewer errors than patients. The findings of our study are consistent with those from previous studies and support the inference that patients with PD experience difficulties in certain cognitive domains, including consolidation of declarative and procedural memory (24, 25, 47).

As patients in our study had been treated for NFPA using stable treatment for at least 1 year before study enrolment, their poor memory performance suggests that cognitive dysfunction may be a feature of NFPA or a limitation of available replacement therapy. Persistent cognitive dysfunction may be due to electrophysiological or neurovascular changes due to the tumour, or patients may suffer from coexistent GH deficiency, which in our setting we are unable to replace due to cost (27). GH replacement therapy has been shown to have positive effects on performance in many cognitive domains, including memory (12, 48). For example, Oertel *et al.* (49) conducted a double-blind controlled trial over 6 months, during which adult GH-deficient patients *n* = 18 were randomised to either a placebo group or a group in which they were treated with recombinant human GH and found that attention improved in GH deficient hypopituitary patients following at least 3 months of GH therapy, in contrast to the placebo group, where no improvement was found.

### Hypothesis 2: within-group comparisons on sleep quality and cognitive function

We hypothesised that both patients and controls would have better memory performance when a period of sleep, rather than a period of wakefulness, separated learning from recall. This prediction was not confirmed; however, in both groups, performance on the LM & FTT was better in the *sleep* than the *wake* condition. A substantial proportion of our patients had both radiotherapy and surgery, and a minority had GH deficiency but were not replaced. Both radiotherapy and GH deficiency could have contributed to poor memory and cognition, but the relative negative impact of each could not be delineated. Although it was expected that GH deficiency and radiotherapy could have exacerbated sleep disturbances in our patients (50, 51), we were unable to demonstrate either objective or subjective differences between patients and controls.

This finding contrasts with a large body of the literature showing that a period of sleep, more than a period of normal waking, benefits the retention of previously learnt declarative material (52, 53) and previously learnt procedural tasks (54, 55).

Sleep is crucial for memory consolidation (56, 57), with a few theories attempting to explain the process of sleep-dependent memory consolidation. These theories suggest that the consolidation of various types of memory is reliant on the characteristics of sleep stages (57), and that the sequence in which one sleep stage follows the other throughout the night is crucial (58). In our study, we invoked the possibility that patients may have experienced sleep which did not facilitate memory consolidation; for example, there was inadequate time in the appropriate sleep stages, and their transitions between sleep stages may have been non-ideal. Our study was limited by not examining the length, depth, and duration of sleep stages.

### Hypothesis 3: between-group comparisons on cognitive tests

Hypothesis 3 stated that a preceding period of sleep would be more beneficial for memory consolidation in HCs than in patients, which was only partially confirmed, as post-sleep testing showed that controls retained significantly more information from the LM subtest than patients. Controls exhibited better post-training FTT performance post-sleep than patients.

Some sleep researchers advocate that sleep efficiency (i.e. the ratio of time spent in bed to time spent sleeping) should be maintained above 85% to yield optimum health benefits (59, 60). In our study, both patients and controls recorded an average of 91% sleep efficiency, with no between-group differences. In contrast, overnight periods of sleep were more beneficial for the cognitive performance of controls than patients. The gross measures of sleep quality (e.g. TST, number of minutes awake after sleep onset, number of awakenings after sleep onset) do not reveal sleep disruptions, which otherwise could have been elicited with more sensitive instruments. Sleep architecture disruptions and the amount of time spent in the critical sleep stages may account for this difference between patients and controls, especially with respect to declarative memory. Previous studies have shown evidence of altered sleep architecture in PD patients, particularly reduced and interrupted time in REM sleep and an increase in stage 1 sleep (20, 61), accounting for declarative memory deficits (62).

### Hypothesis 4: within-group associations between sleep and cognitive variables

In the patient group, our analyses suggested that better sleep efficiency was associated with a smaller post-training error rate on the FTT. In the HC group, analyses suggested that fewer awakenings were associated with better post-training performance on the FTT. However, analyses detected no other significant associations in the control or patient group, meaning the current findings do not support the wealth of research showing that sleep plays a vital role in cognitive function (55, 56, 57, 58).

### Limitations and directions for future research

Data for this study were collected during the COVID-19 pandemic (2020–2022) and thus are reliant on observed associations but limited by participant numbers and possibly being underpowered. Our device used to record objective sleep data may be limited due to it being insensitive to discern sleep stages throughout the night. Our study did not sample cortisol concentrations, which may have generated data relating to memory, cognitive deficits, and timing of dosing of hydrocortisone replacement therapy. We did not ascertain how long patients were awake before being assessed, thus the duration of wakefulness may have differed, which could have influenced the results of this study. The majority of our group of patients had undergone the combination of radiotherapy and surgery, and a smaller proportion had unreplaced GH deficiency, representing a heterogeneous group of patients, limiting our ability to attribute a single factor to poorer cognitive performance, compared with HCs. Our patients differed from the remaining group in respect of the frequency of radiotherapy (70%) versus 16% in the remainder of the cohort, limiting its generalisability. This could have arisen through random error and may have resulted in bias and imprecision. The large proportion who underwent radiotherapy may have influenced cognitive performance and memory negatively. In addition, because we gained information about our patients from a database, we could not obtain details about the characteristics of the adenoma at diagnosis, the type of radiotherapy, and the follow-up time between treatment and evaluation. To eliminate the possibility of the practice effect, different forms of the LM and the FTT were used in the sleep and wake conditions. As different versions of the neuropsychological tests were used, we believe that a period of 1 week between conditions may have been sufficient; however, it may have been preferable to separate the studies by a period of 3–6 weeks to more confidently minimise any residual effects of prior testing.

## Summary and conclusion

We aimed to advance the understanding of psychological functioning in patients with PD, focusing on understanding the influence of sleep on memory in patients with NFPA, and to investigate whether sleep enhances memory consolidation in patients, as it does in HCs. Significantly poorer cognitive performance in patients than in matched HCs was identified, but there were no significant differences in sleep quality. A period of sleep (rather than a period of normal waking) between learning and recall was advantageous for performance on certain cognitive tasks (e.g. LMT, FTT) in HCs compared with NFPA patients, raising the question as to whether NFPA patients experience cognitive difficulties as a result of disrupted sleep architecture (e.g. the amount of time spent in the particular sleep stages) versus the amount of time spent asleep. This would require much more granular investigation of sleep architecture to discern these differences using polysomnographic techniques.

Although our a *priori* hypotheses were only partially confirmed, our findings contribute to the existing body of psychological research on PD patients and may provide an impetus for further research in the field. Potential clinical and practical implications are that patients with PD may, for instance, lead to a focus on specific memory rehabilitation interventions to improve cognition and memory. These interventions may assist in improving their capacity to complete important daily activities and to participate in the normal labour force, which requires cognitive ability.

## Declaration of interest

The authors declare that there is no conflict of interest that could be perceived as prejudicing the impartiality of the work reported.

## Funding

This work did not receive any specific grant from any funding agency in the public, commercial, or not-for-profit sector.

## Patient consent

Consent has been obtained from each patient after a full explanation of the purpose and nature of all procedures used.
